# Shugoshin 2 is a biomarker for pathological grading and survival prediction in patients with gliomas

**DOI:** 10.1038/s41598-021-97119-4

**Published:** 2021-09-17

**Authors:** Ying Kao, Wen-Chiuan Tsai, Ssu-Han Chen, Shao-Yuan Hsu, Li-Chun Huang, Chih-Ju Chang, Shih-Ming Huang, Dueng-Yuan Hueng

**Affiliations:** 1 Graduate Institute of Medical Sciences, National Defense Medical Center, Taipei, Taiwan, ROC; 2 Division of Neurosurgery, Department of Surgery, Taipei City Hospital Zhongxing Branch, Taipei, Taiwan, ROC; 3 University of Taipei, Taipei, Taiwan, ROC; 4 Department of Pathology, Tri-Service General Hospital, National Defense Medical Center, Taipei, Taiwan, ROC; 5 Division of Neurosurgery, Department of Surgery, Taipei City Hospital, Renai Branch, Taipei, Taiwan, ROC; 6 Department of Biochemistry, National Defense Medical Center, Taipei, Taiwan, ROC; 7 Division of Neurosurgery, Department of Surgery, Cathay General Hospital, Taipei, Taiwan, ROC; 8 Department of Medicine, School of Medicine, Fu Jen Catholic University, New Taipei City, Taiwan, ROC; 9 Department of Mechanical Engineering, National Central University, Taoyuan County, Taiwan, ROC; 10 Department of Neurological Surgery, Tri-Service General Hospital, National Defense Medical Center, Taipei, Taiwan, ROC

**Keywords:** Cancer, Cell biology, Genetics, Molecular biology, Neuroscience, Biomarkers, Neurology, Oncology, Pathogenesis, Risk factors

## Abstract

Glioblastomas are the most common type of adult primary brain neoplasms. Clinically, it is helpful to identify biomarkers to predict the survival of patients with gliomas due to its poor outcome. Shugoshin 2 (*SGO2*) is critical in cell division and cell cycle progression in eukaryotes. However, the association of *SGO2* with pathological grading and survival in patients with gliomas remains unclear. We analyzed the association between *SGO2* expression and clinical outcomes from Gene Expression Omnibus (GEO) dataset profiles, The Cancer Genome Atlas (TCGA), and Chinese Glioma Genome Atlas (CGGA). *SGO2* mRNA and protein expression in normal brain tissue and glioma cell lines were investigated via quantitative RT-PCR, Western blot, and IHC staining. The roles of *SGO2* in proliferation, migration, and apoptosis of GBM cells were studied with wound-healing assay, BrdU assay, cell cycle analysis, and JC-1 assay. The protein–protein interaction (PPI) was analyzed via Search Tool for the Retrieval of Interacting Genes/Proteins (STRING). *SGO2* mRNA expression predicted higher grade gliomas than non-tumor brain tissues. Kaplan–Meier survival analysis showed that patients with high-grade gliomas with a higher *SGO2* expression had worse survival outcomes. *SGO2* mRNA and protein expression were upper regulated in gliomas than in normal brain tissue. Inhibition of *SGO2* suppressed cell proliferation and migration. Also, PPI result showed SGO2 to be a potential hub protein, which was related to the expression of AURKB and FOXM1. *SGO2* expression positively correlates with WHO pathological grading and patient survival, suggesting that *SGO2* is a biomarker that is predictive of disease progression in patients with gliomas.

## Introduction

The most common form of primary intrinsic brain tumor in adult patients is glioma. There are approximately 16,000 new cases of gliomas every year in the US^1^. As the World Health Organization (WHO) classification^2^, glioma is classified into four grades, among which glioblastoma multiforme (GBM), WHO grade IV, is the most malignant central nervous system tumor. However, GBM is the most frequent primary brain tumor in adult patients, accounting for 54% of all adult patients^1^. According to the guideline from National Comprehensive Cancer Network (NCCN)^3^, the standard treatment strategies for GBM are maximal safe resection, radiation therapy, and temozolomide (TMZ) treatment, as the protocol mentioned by Stupp et al.^4^. Bevacizumab, a monoclonal antibody for endothelial growth factor receptors, can be administered to patients with tumor recurrence^5–8^. However, the overall survival rate of patients with GBM is approximately 14.6 months^4^. In addition to its histological characteristics, several genetic factors, such as IDH-1/2 mutation, have been added to the diagnosis of GBM because of their influences on treatment and prognosis^3, 9^. Thus, identifying new biomarkers that relate to the development, differentiation, and recurrence of GBM to provide new directions in diagnosis and treatment will be a major focus of future studies on GBM.

Shugoshin 2 (*SGO2*), which is a conserved centromeric protein belonging to the Shugoshin family, plays an important role during cell division in eukaryotes^10^. *SGO2* functions as a guardian that protects centromeric cohesion from precocious dissociation, resulting in an early separation of sister chromatids, via the Shugoshin–serine/threonine protein phosphatase 2A (PP2A) interaction^11^. During meiosis, *SGO2* maintains normal gametogenesis by preventing the premature release of REC8- cohesin complex from the centromere^12^. Huang et al. have found that *SGO2* is critical for the correct attachment of kinetochore to the centromere and that *SGO2*-deficient cells are defective in kinetochore attachment, which results in lagging chromosomal formation during anaphase^13^.

In this study, we hypothesized that *SGO2* is overexpressed in patients with high-grade gliomas. First, we investigated the relationship between *SGO2* expression and survival in patients with gliomas and attempted to investigate the association of *SGO2* expression with WHO pathological grading of human gliomas. Then, the Gene Expression Omnibus (GEO) dataset profiles, The Cancer Genome Atlas (TCGA), Chinese Glioma Genome Atlas (CGGA), RT-PCR, and Western blotting analysis suggested that *SGO2* might be a new prognostic biomarker for human gliomas. Further, we explored the biological role of *SGO2* in glioma cell migration, proliferation, apoptosis, and protein–protein interaction. These resulted indicated that *SGO2* has potential to be the target for new treatment design.

## Materials and methods

### SGO2 gene expression, survival outcome, and pathological grading in human gliomas

The methodology for the analyses of functional genomic databases was as previously described^14–16^. In brief, 100 sheets of de-linked data (GDS1816/230165_at/SGO2) on *SGO2* mRNA expression, sex, age, pathologic grading, and survival rates of patients with primary high-grade gliomas were obtained from NCBI (available online: https://www.ncbi.nlm.nih.gov/geo/tools/profileGraph.cgi?ID=GDS1816:230165_at). Twenty-three sheets of data without detailed information on age and survival times were excluded; thus, a total of 77 sheets were included in the statistical analyses. An additional database (GDS1962/230165_at/SGO2) that contained 180 sheets from 81 patients with grade IV gliomas, 19 with grade III gliomas, seven with grade II gliomas, 23 without tumors (non-tumor control) (Available online: https://www.ncbi.nlm.nih.gov/geo/tools/profileGraph.cgi?ID=GDS1962:230165_at) and included. 38 with grade II oligodendroglioma and 12 with grade III oligodendroglioma were excluded. Also, we analyzed the The Cancer Genome Atlas (TCGA), and Chinese Glioma Genome Atlas (CGGA, http://www.cgga.org.cn)^17^ database to obtain the glioma overall survival and gene expression. The TCGA dataset was acquired through the cBio Cancer Genomics Portal (http://cbioportal.org)^18^, which containing 343 patients with gliomas included 61 panels of grade II gliomas, 130 panels of grade III gliomas, and 152 panels of grade IV gliomas. The CGGA dataset comprised 211 patients with 75 panels of grade II gliomas, 28 panels of grade III gliomas, and 108 panels of grade IV gliomas.

The Kaplan–Meier method was used to analyze the overall survival rates and cohorts of low- vs. high‐*SGO2* expressions in high‐grade gliomas from the GEO profile (GDS1816/230165_at/SGO2), TCGA, and CGGA. *SGO2* expression cutoff point was decided using statistical analysis. The GraphPad Prism 5 software was used to generate the figures, and P < 0.05 was defined as statistical significance.

### Cell culture and RNA interference

LN229, U118MG and U87MG cell lines were purchased from American Type Culture Collection (ATCC). GBM8401 glioma cell line was commercially available and obtained from Bioresource Collection and Research Center (BCRC number 60163, Hsinchu, Taiwan). LN229 and GBM8401 cells were harvested in Dulbecco’s modified Eagle’s medium (DMEM) containing 2% fetal bovine serum (FBS), penicillin, and streptomycin. U87MG cells were maintained in Dulbecco’s modified Eagle’s medium containing 10% FBS, penicillin, and streptomycin. U118MG were maintained in DMEM containing 10% FBS, penicillin, and streptomycin. All these cells were maintained at 37 °C and 5% CO_2_. RNA interference was performed according to previous description^19–21^. In brief, LN229 and GBM8401 cells were transfected 24 h post-culture with *SGO2* small interfering RNA (siRNA) (siGENOME SMARTpool, Dharmacon) at final 25 nM in antibiotic-free media using DharmaFECT Transfection Reagent 1 (Dharmacon) following the manufacturer’s instructions. Non-targeting siRNA (siGENOME Non-Targeting siRNA Control Pool #1, Dharmacon) was used as negative control.

### RNA isolation and quantitative RT-PCR

Total RNA was extracted using EasyPure Total RNA reagent (Bioman, Taipei, Taiwan) according to the manufacturer’s protocol. For cDNA synthesis, 1.0 μg RNA was reverse transcribed into cDNA using Oligo dT primer with MMLV Reverse Transcriptase (Epicentre Biotechnologies, Madison, WI, USA). Normal brain cDNA was purchased from Origene Technologies (Rockville, MD, USA).

Gene expression was quantified using quantitative RT-PCR (qRT-PCR) and performed in an illumina ECOTM Real-Time PCR system. Amplifications were performed using an IQ2 fast qPCR system with ROX (Bio-genesis Technology Inc., Taipei, Taiwan). Relative quantitative gene expression against an internal control, GAPDH, was performed using the 2^−ΔΔCt^ method^22^. The primer pairs used were *SGO2* forward, 5′-ATGTGGTGCATGGCCTAAAAA-3′ and reverse, 5′-GGGGTACATATTGGTGATCTGC-3′ and GAPDH forward, 5′-GCACCGTCAAGGCTGAGAAC-3′ and reverse, 5′-ATGGTGGTGAAGACGCCAGT-3′.

### Cell lysate preparation and Western blot

Cells were lysed using RIPA buffer (100 mM Tris–HCl, 150 mM NaCl, 0.1% SDS, and 1% Triton-X-100) at 4 °C for 10 min, and cell lysates were harvested by centrifugation at 15,000 rpm for 10 min to obtain the supernatants. Normal brain lysates were purchased from Origene Technologies. Thirty-microgram cell lysates from each group were applied to 10% sodium dodecyl sulfate polyacrylamide gel electrophoresis. Proteins were transferred onto polyvinylidene fluoride membranes (Millipore, MA, USA) and blocked with 5% skim milk in TBST for 1 h at room temperature. Anti-SGO2 antibody (Atlas Antibodies AB, Stockholm. Sweden) was diluted at a ratio of 1:1000 with SignalBoost Immunoreaction Enhancer Kit following the protocol of manufacturer. Band were detected using enhanced chemiluminescence and X-ray film (GE Healthcare, Piscataway, NJ, USA).

### Analysis of the immunohistochemical (IHC)staining of human gliomas specimen

IHC staining was conducted with commercially available tissue microarrays (BS17015a and NGL961; Biomax, Rochester, NY, USA) according to previous protocol^23, 24^. The tissue microarrays were incubated with a polyclonal rabbit anti-human SGO2 antibody (HPA035163, Atlas Antibodies, Stockholm. Sweden) which was diluted in phosphate buffered saline (PBS) at a ratio of 1:20 for 1 h at room temperature, washed 3 times (each for 5 min in PBS), incubated with biotin-labeled secondary immunoglobulin (1:100, DAKO, Glostrup, Denmark) for 1 h at room temperature, washed 3 times, and treated with 3-amino-9-ethylcarbazole substrate chromogen (DAKO) at room temperature to visualize peroxidase activity^16^. Labeling index was scored accordance with multiplying quantity by intensity. The quantity was defined as Negative: 0, < 25%: 1, 25–75%: 2, and > 75%: 3 The intensity was defined as Negative: 0, Weak: 1, Moderate:2, and Strong: 3^25^.

### Cell proliferation, cell cycle analysis, cell apoptosis, and flow cytometry analysis

For cell counting assay, we seeded LN229 and GBM8401 cells (2.5 × 10^4^ per well) in a 12-well plate. Cells were transfected with 25 nM siRNA on the next day. The cells were counted at 24, 48, and 72 h after transfection. Before counting, cells were mixed with trypan blue for 3–5 min according to the previous description^26^. The differences in growth rate between the experimental groups and the control groups was detected in five independent experiments. For cell proliferation analysis, LN229 and GBM8401 cells were transfected with siRNA then processed with the FITC-BrdU Flow Kits according to the manufacturer’s instructions (BD Biosciences). To analyze the distribution of cell cycle stage after RNA interference, we detected the DNA content by fluorescence activated cell sorting (FACS) as previous description^27^. The cells in experimental and control group were fixed in 70% ethanol at 4 °C and kept at − 20 °C overnight. Then the cells were washed twice with cold phosphate-buffered saline (PBS), and stained with propidium iodide (PI) solution (50 μg/ml PI in PBS, 1% Tween 20 and 10 μg/ml RNase A) for 30 min in the dark. Then the DNA content was analyzed by fluorescence activated cell sorting (BD Biosciences, San Jose, CA, USA) in two independent experiments. For apoptosis, we used JC-1 assay accordance with previous description^28–30^. Briefly, cells were seeded in 6-well plate then transfected with si*SGO2* or siControl on next day. Cells were collected to proceed the protocol as the manufacturer’s guide (BM MitoScreen). All these samples were analyzed by FACSCalibur flow cytometer (BD Biosciences) and Cell Quest Pro software (BD Biosciences).

### Cell migration assay

We used wound-healing assay for cell migration analysis according to previous studies^31, 32^. In brief, LN229 and GMB8401 (2 × 10^5^) were seeded into 12-well plates and grown at 37 °C in a 5% CO_2_ incubator. RNA interference was performed on the next day. On the day 3, we removed the medium when cell confluence reached 90% and made a wound in the monolayer with a pipette tip. Then, we washed the plate for three times to remove the non-adherent cells. The wound area was photographed immediately after wounding (0 h) and at 16 h post wounding. The migration rates were computed according to the change of wound area measured by ImageJ software (NIH, Bethesda, MD).

### Protein–protein network and signaling pathways analysis

Known and predicted protein–protein interactions were analyzed using the Search Tool for the Retrieval of Interacting Genes/Proteins (STRING) database version 10.0 (http://string-db.org)^33^.

### Statistical analysis

To analyze *SGO2* expression in different pathological grades from the dataset (GDS1962/230165_at/SGO2, TCGA, and CCGA), we used a single tailed t-test. The R 3.0.1 software (R Foundation for Statistical Computing, Vienna, Austria) package for the Bonferroni method was used to adjust the p-value to eliminate the risk of type I error during multi‐group analyses.

### Ethical approval

The study protocol was approved by the Institutional Review Board (TSGHIRB No.: 2-105-05-052) of Tri-Service General Hospital.

## Results

### SGO2 expression positively correlates with WHO grading of human gliomas

**Figure 1:**
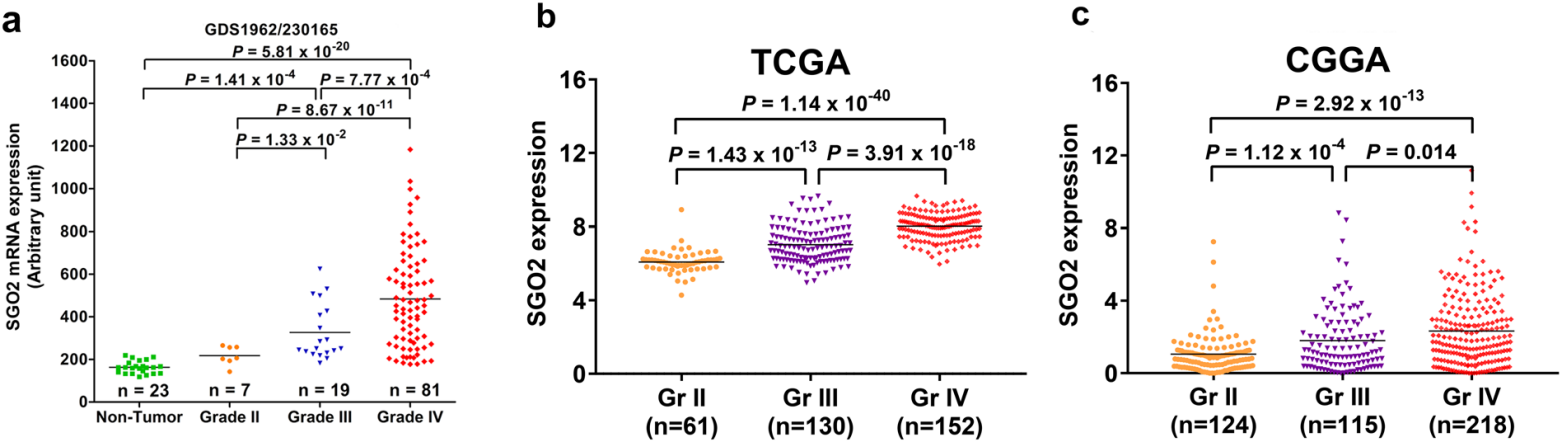
The expression level of *SGO2* is related to pathological grading of gliomas. The *SGO2* mRNA level in different grade of gliomas and non-tumor brain tissue from GEO profile (GDS1962/230165_at/SGO2) (**a**), TCGA (**b**), and CCGA (**c**). *SGO2* expression was significantly higher in high-grade gliomas (Grade III and IV) than in low-grade gliomas (Grade II) and non-tumor control. The Y-axis indicates the *SGO2* mRNA expression. The *p* value was adjusted by Bonferroni method in R software (version 3.0.1) between each group.

In Fig. 1a, the *SGO2* mRNA expression level was significantly higher in the WHO grade IV (*n* = 81) than in grade III gliomas (*n* = 19; *p* = 7.77 × 10^–4^), grade II gliomas (*n* = 7; *p* = 8.67 × 10^−11^), and in non-tumor controls (*n* = 23; *p* = 5.81 × 10^−20^). Also, *SGO2* expression was higher in WHO grade III gliomas than in grade II gliomas (*p* = 0.0133) and in non-tumor control (*p* = 1.41 × 10^–4^, *p* adjusted by Bonferroni method).

In TCGA dataset, the *SGO2* mRNA expression level was significantly higher in the WHO grade IV (*n* = 152) than in grade III gliomas (*n* = 130; *p* = 3.91 × 10^–18^) and in grade II gliomas (*n* = 61; *p* = 1.14 × 10^−40^). Moreover, the *SGO2* mRNA expression was grater in WHO grade III gliomas then in grade II gliomas (*p* = 1.43 × 10^–13^, *p* adjusted by Bonferroni method) (Fig. 1b).

In CGGA data shown in Fig. 1c, the *SGO2* mRNA expression level was significantly higher in WHO grade IV (*n* = 218) than in grade III gliomas (*n* = 115; *p* = 0.014) and in grade II gliomas (n = 124; *p* = 2.92 × 10^−13^). Also, we found that the *SGO2* expression was greater in WHO grade III gliomas then in grade II gliomas (*p* = 1.12 × 10^−4^, *p* adjusted by Bonferroni method). These three independent cohort data analyses suggested that high grade gliomas were correlated with *SGO2* overexpression.

### SGO2 expression correlates with poor survival in high-grade gliomas

**Figure 2:**
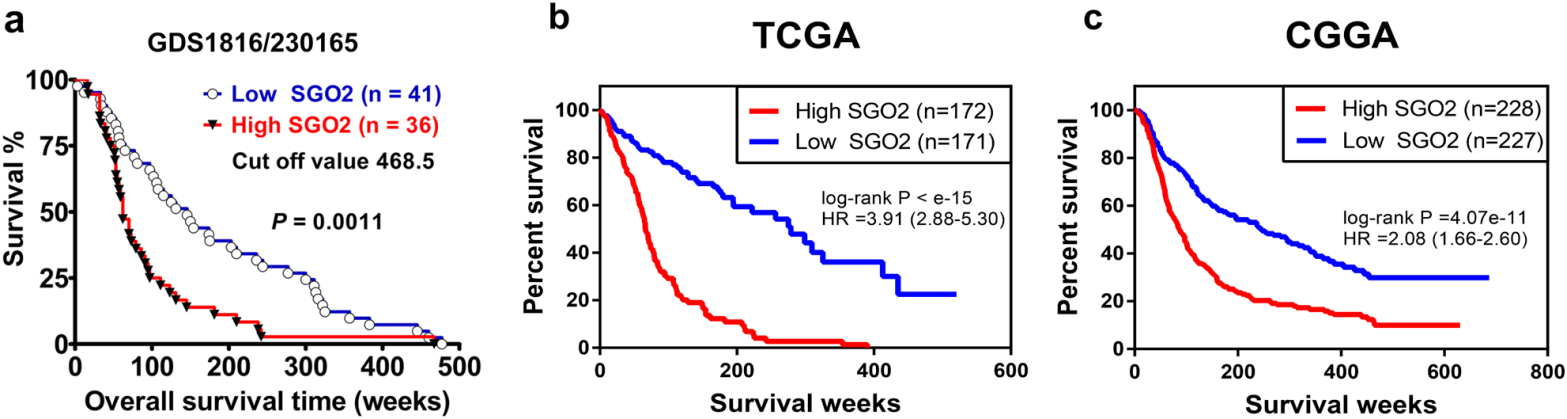
The expression level of *SGO2* is related to the survival of patients with high-grade gliomas. The Kaplan–Meier survival curve analyzed from GEO profile (GDS1816/230165_at/SGO2) (**a**), TCGA (**b**), and CGGA (**c**). Data showed that patients with high expression of *SGO2* had unfavorable survival outcome. (GDS1816/230165_at/SGO2, *n* = 77, *p* = 0.0011 by log-rank test, 95% CI 1.000–1.00, hazard ratio 1.001; TCGA, *n* = 343, *p* < 1 × 10^–15^ by log-rank test, 95% CI 0.87–5.30, hazard ratio 3.90; CGGA, *n* = 209, *p* = 1.23 × 10^–11^ by log-rank test, 95% CI 2.13–4.25, hazard ratio 3.01).

The Kaplan–Meier survival analysis shown in Fig. 2a revealed that elevated *SGO2* mRNA expression related to an unfavorable survival in patients with high‐grade glioma (*n* = 77, *p* = 0.0011, by log‐rank test; 95% confidence interval:1.000–1.001, hazard ratio 1.001). Moreover, two larger sample size data set, shown in Fig. 2b,c, suggested that *SGO2* overexpression correlated with poor survival outcome in high grade glioma patients with statistical significance (TCGA, *n* = 343, *p* < 1 × 10^–15^ by log-rank test, 95% CI 2.88–5.30, hazard ratio 3.91; CGGA, *n* = 455, *p* = 4.07 × 10^–11^ by log-rank test, 95% CI 1.66–2.60, hazard ratio 2.08).

### SGO2 mRNA and protein expression is increased in human glioma cells

**Figure 3:**
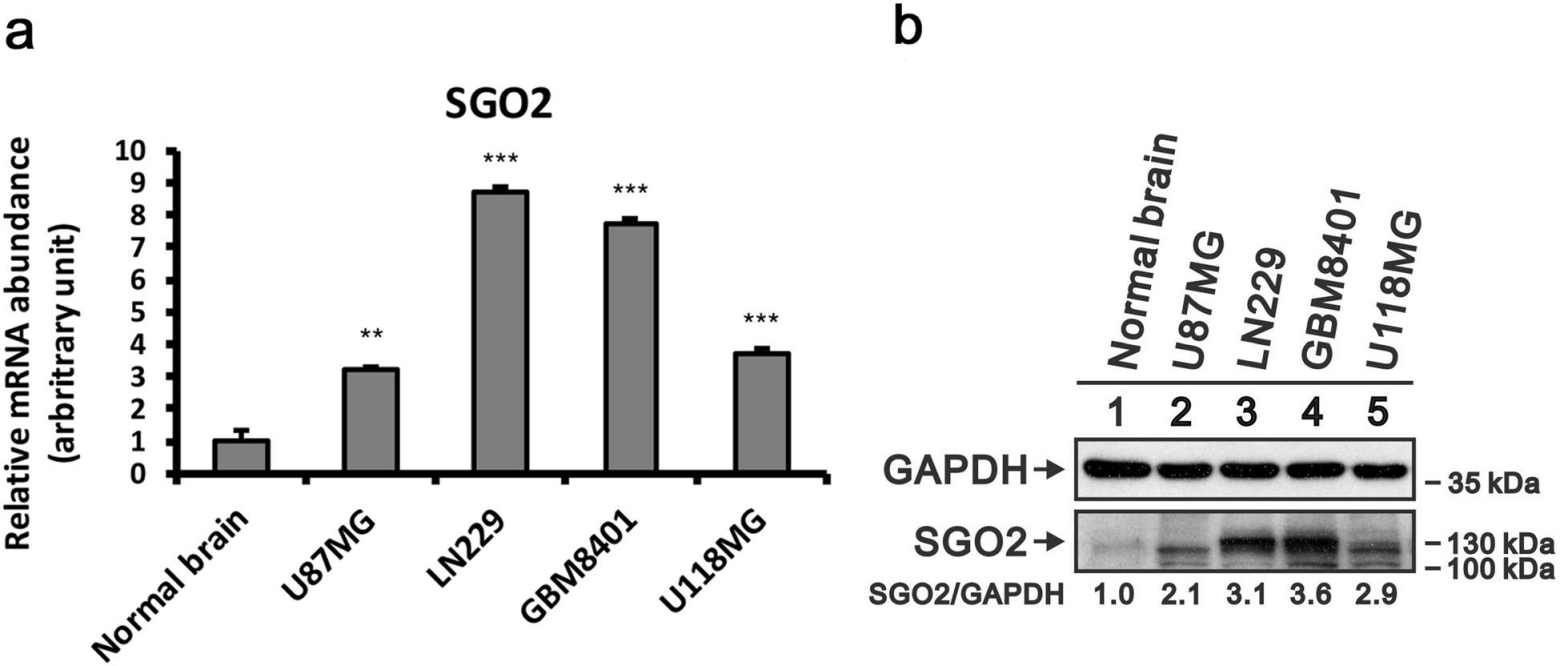
Validation of *SGO2* mRNA and protein levels in glioma cell lines and normal brain tissue (**a**) qRT-PCR was performed to examine *SGO2* mRNA expression and the quantitative results are shown in glioma cell lines. The relative expressions were normalized with normal brain. Bars mean ± SEM; *p < 0.05, **p < 0.01, ***p < 0.005 showed significant differences. Data are representative of three independent experiments. (**b**) Protein lysates of glioma cell lines, including U87MG, LN229, GBM8401, and U118MG were applied to SDS-PAGE and Western blot analysis to quantitate SGO2 protein expression (full length blot is presented in Supplementary Fig. 1). GAPDH served as a loading control.

We further investigated the expression of *SGO2* mRNA amount normal brain, WHO grade IV glioma cell lines including LN229, U87MG, GBM8401, and U118MG. The results as showed in Fig. 3a, revealed that the expression of *SGO2* was significantly increased in glioma cells comparing with normal brain tissue. Using western blot, we found the expression of *SGO2* protein revealed higher in LN229 and GBM8401 (Fig. 3b) then in normal brain.

### SGO2 protein expression is increased in human high-grade gliomas

**Figure 4:**
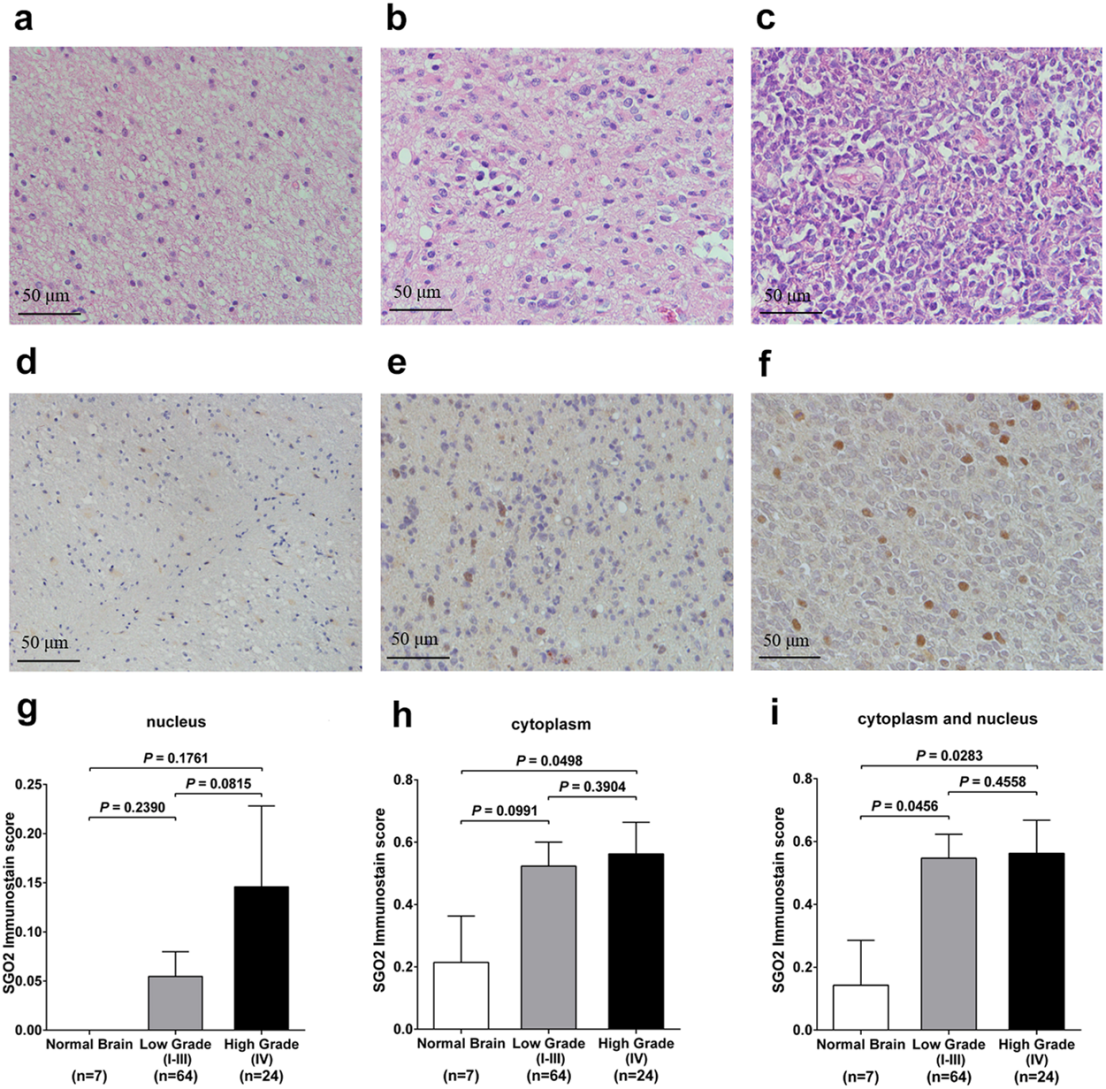
Validation of SGO2 protein expression in human gliomas and non-tumor brain tissue. Hematoxylin and eosin staining of non-tumor brain tissue (**a**), low grade (**b**) and high grade gliomas (**c**). The immunohistochemical staining of SGO2 on non-tumor brain tissue (**d**), low grade (**e**), and high grade gliomas (**f**) (scale bar: 50 μm). (**g**–**i**) The SGO2 immunostaining scores in normal brain tissue, low-grade glioma and high-grade glioma were statistically analyzed. The adjusted *p* value was calibrated between each group.

To investigate the SGO2 protein expression in non-tumor brain tissues and human gliomas tissues, IHC staining of two human tissue microarrays were conducted (Fig. 4a–f). We found that the immunohistochemical staining score of SGO2 was higher in high-grade (WHO IV) gliomas than in low-grade (WHO grade I, II, and III) gliomas (nucleus: *p* = 0.0815; cytoplasm: *p* = 0.3904; cytoplasm and nucleus: *p* = 0.4558). Also, the SGO2 immunohistochemical staining score was higher in high-grade gliomas then in normal brain (nucleus: *p* = 0.1761; cytoplasm: *p* = 0.0498; cytoplasm and nucleus: *p* = 0.0283, respectively). Moreover, SGO2 immunostain score was higher in low-grade gliomas than in normal brain (nucleus: *p* = 0.2390; cytoplasm: *p* = 0.0991; cytoplasm and nucleus: 0.0456, *p* adjusted by Bonferroni method, Fig. 4g–i). The result suggested that SGO2 protein overexpression in high-grade gliomas compared with non-tumor brain tissues.

### SGO2 down regulation inhibits cell proliferation in glioma cells

**Figure 5:**
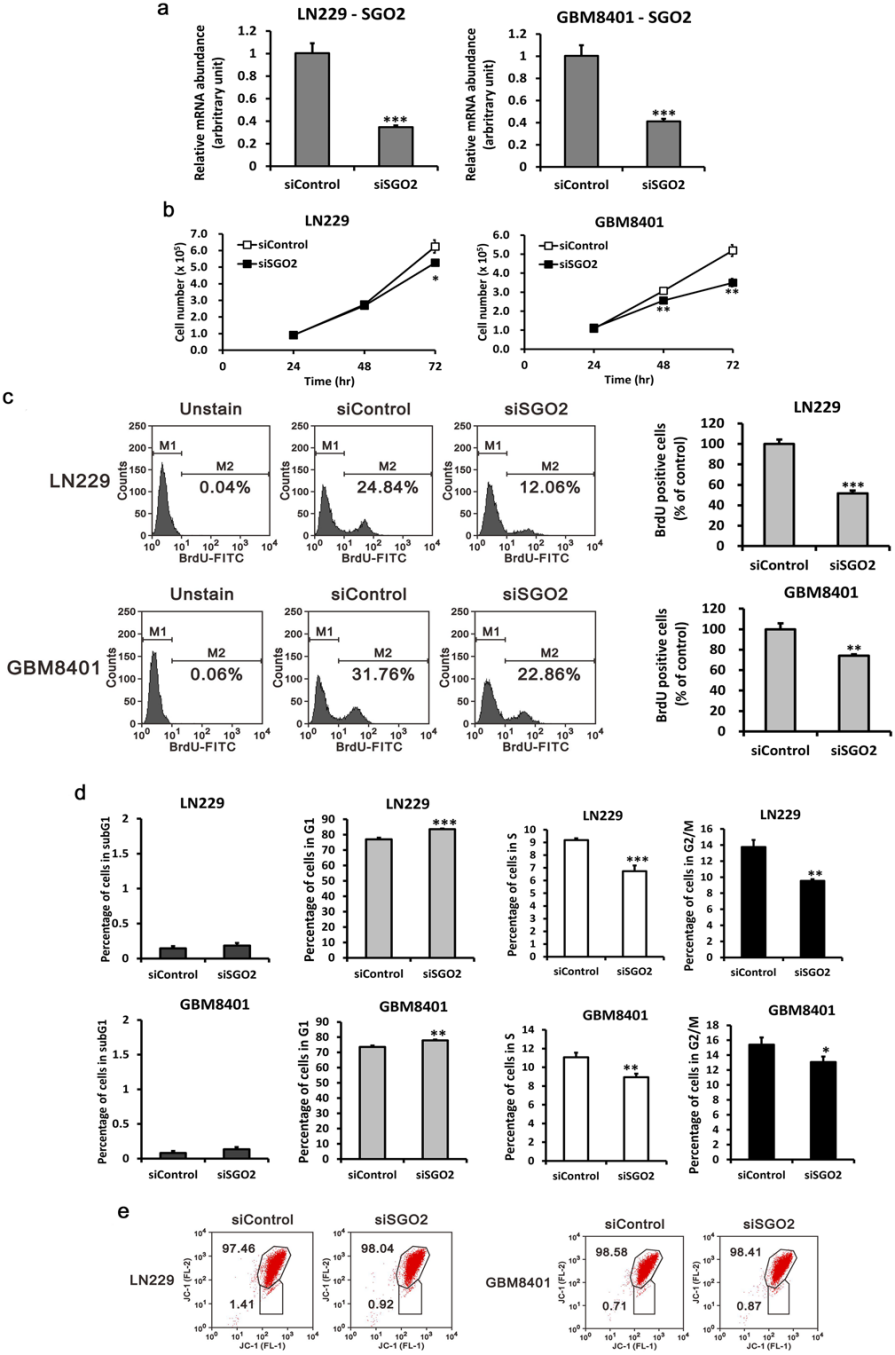
The effect of *SGO2* on cell proliferation and apoptosis (**a**) The *SGO2* knockdown model constructed by siRNA 25 nM transfection into LN229 and GBM8401 cell lines. The knockdown efficiency of *SGO2* siRNA or control siRNA in infected LN229 and GBM8401 cells measured by RT-qPCR. Bars, mean ± SEM; *p < 0.05, **p < 0.01, ***p < 0.005 showed significant differences. Data are representative of three independent experiments. (**b**) LN229 and GBM8401 cells were transfected with 25 nM siRNA or siControl. Cell count was determined at the indicated time points. The data are expressed as the mean ± s.d.; n = 3; **p < 0.01, and ***p < 0.001. (**c**) LN229 and GBM8401 cell with si*SGO2* or siControl transfection were labeled with BrdU then proceeded analysis by flow cytometry (**p < 0.01, ***p < 0.005). (**d**) Cell cycle analysis of LN229 and GBM8401 si*SGO2* cells was determined by propidium iodide (PI) stain and flow cytometry. The data are expressed as the mean ± s.d.; n = 3; *p < 0.05, **p < 0.01. (**e**) Cell apoptosis analysis of LN 229 and GBM8401 si*SGO2* cells were determined by tetraethylbenzimidazolylcarbocyanine iodide (JC-1) dye and flow cytometry.

To explore the effect of *SGO2* in glioma tumorigenesis, we used siRNA to knock down *SGO2* expression in LN229 and GBM8401 cells (Fig. 5a). *SGO2* has been reported to protect centromeric cohesion during cell division^34^. Thus, we investigated the effect of *SGO2* in glioma cell proliferation. Cell counting of LN229 and GBM8401 decreased after *SGO2* down regulation (Fig. 5b). Using BrdU assay, we found that *SGO2* knockdown can resulted in decreased the proportion of active cell proliferation compared with siControl glioma cells (Fig. 5c). Furthermore, cell cycle analysis showed that *SGO2* down regulation leaded to G1 phase arrest in LN229 and GBM8401 cells (Fig. 5d). Then we further investigate the relationship between *SGO2* and cell apoptosis. In JC-1 assay, we found that the proportion of cell apoptosis revealed no different between si*SGO2* and siControl glioma cells (Fig. 5e). Based on these results and the biological function of *SGO2*, we believe that *SGO2* may have crucial role in glioma cells proliferation.

### SGO2 plays an important role in glioma cell migration

**Figure 6:**
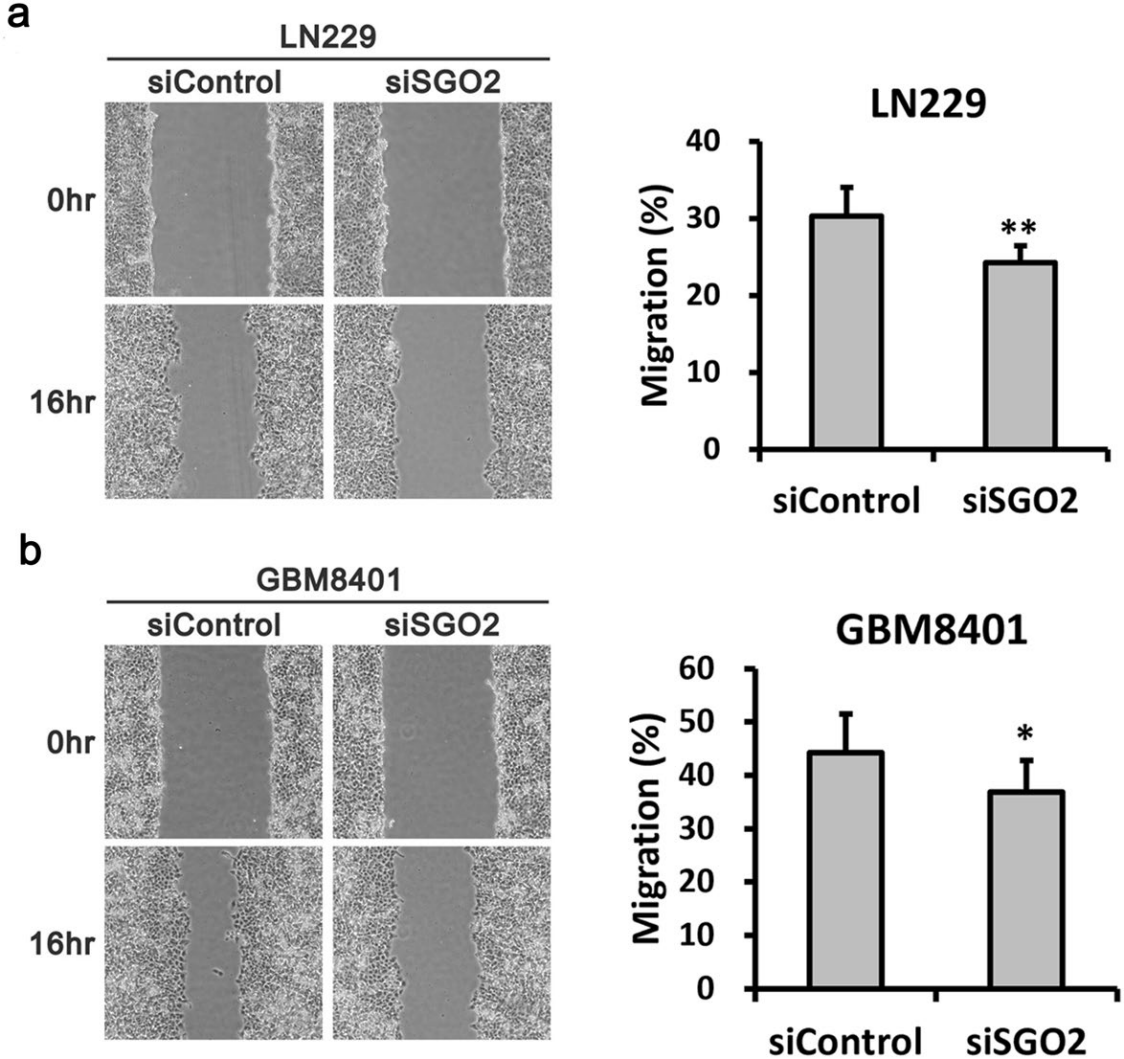
The effect of *SGO2* knockdown on cell migration detected by wound-healing assays. Images and Quantitative analysis of LN229 (**a**) and GBM8401 (**b**) cells in the wound-healing assay. Data are presented as the mean ± SD (n = 3). *p < 0.05, **p < 0.01.

To investigate effect of *SGO2* in glioma cell migration, we performed wound healing and migration assays. The results showed that the ability of LN229 and GBM8401 cell migration revealed significant decreased in si*SGO2* compared with siControl. (Fig. 6a,b).

### SGO2 hubs the protein–protein interactions

**Figure 7:**
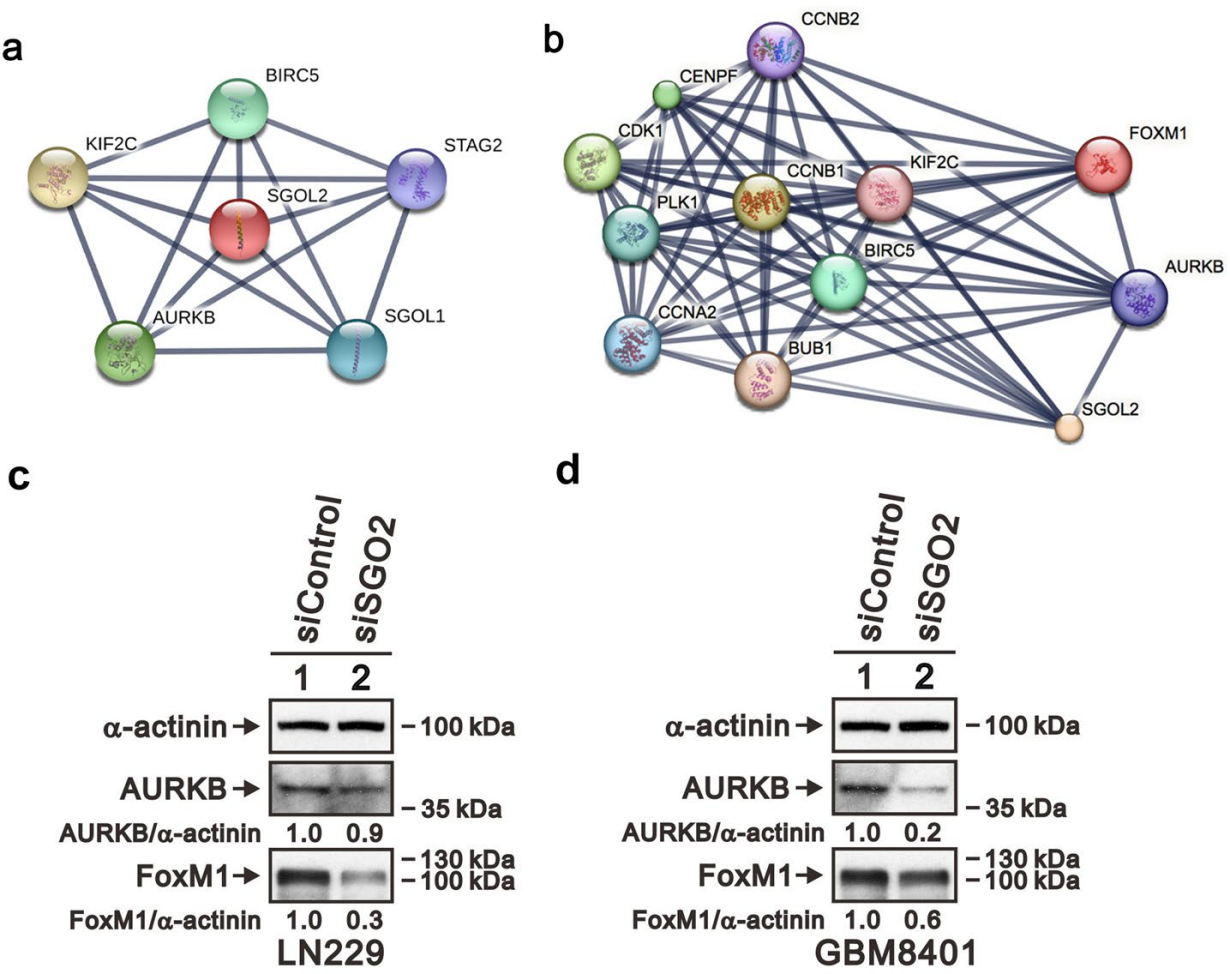
The SGO2 protein–protein interaction (PPI) network. (**a**) In the PPI network established by STRING dataset, SGO2 is a hub protein. (**b**) The STRING dataset also predicted the association between SGO2, ARUKB, and FOXM1. (**c**) Protein lysates of LN229 and GBM8401 were applied to SDS-PAGE and Western blot to investigate the protein expression of AURKB and FOXM1(full length blot is presented in Supplementary Fig. 2). α-actinin served as a loading control.

To further understand the protein–protein interaction (PPI) network of SGO2-regulated oncogenesis, we use Search Tool for the Retrieval of Interacting Genes/Proteins (STRING) database. The network showed that SGO2 had interactions with Aurora B kinase (AURKB) and BUB1 (Fig. 7a,b). Also, SGO2 may have relationship in FOXM1 regulation (Fig. 7b). Further we investigated the expression of AURKB and FOXM1 by Western blot (Fig. 7c,d). The data showed that AURKB and FOXM1 revealed decreased protein expression after SGO2 knockdown, which indicated that SGO2 hubs may the protein–protein interaction.

## Discussion

Till date, no studies have investigated the role of *SGO2* in GBM. This is the first study to investigate *SGO2* expression according to the WHO pathological grading of human gliomas and the association between *SGO2* expression and clinical outcomes. In this study, we found a significantly higher *SGO2* expression in patients with high-grade gliomas than in non-tumor brain tissue controls. We also found that high *SGO2* expression predicts poor survival outcomes in patients with gliomas. Furthermore, *SGO2* overexpression in high grade gliomas was confirmed using qRT-PCR, Western blot, and IHC staining. Together with these results, we thought that *SGO2* has the potential to be a new biomarker for clinical specialists to predict survival outcomes in patients with GBM.

According to the analysis of TCGA database from the Human Protein Atlas (HPA), SGO2 is not considered prognostic in GBM (https://www.proteinatlas.org/ENSG00000163535-SGO2/pathology). However, the analysis of TCGA in HPA only enrolled the cases of GBM (n = 153) but not that of low-grade gliomas (LGG). When analyzed the TCGA database, we enrolled both cases of GBM and LGG so that the n = 343 (grade 2: n = 61, grade 3: n = 130, grade 4: n = 152). Although the case number of GBM is different in our dataset (n = 152) and in HPA dataset (n = 153), but we thought that this is the inter-databased difference. Our result showed that *SGO2* has prognostic value in gliomas.

An accurate chromosomal segregation results in proper cell division. The key processes involved in chromosomal segregation are the formation of sister chromatid cohesion, correct assemblage of spindle, and well linkage between sister kinetochores and microtubules^35–37^. SGO2 has been found to be associated with centromeric cohesion protection and chromosome alignment. SGO2 can help chromosomal passenger complex loading on to centromere during the M phase^38^. Huang et al. have found that SGO2 can recruit mitotic centromere-associated kinesin, which is a microtubule depolymerase, at centromeres to modify the microtubule dynamics, and *SGO2*-deficient HeLa cells revealed chromosome steady during anaphase^13^. These results suggest an important role of *SGO2* in cell division and proliferation. Further investigations of the association of *SGO2* with glioma cell division will substantiate this hypothesis.

Tanno et al. have proposed that Aurora B-induced phosphorylation of SGO2 recruits PP2A and MACK to centromeres^39^. The expression of Aurora B kinase is related to poor clinical survival outcomes in patients with GBM^40^. The inhibition of Aurora A and B kinase can enhance the sensitivity to temozolomide and radiotherapy in glioblastoma cell lines^41^. Furthermore, malignant human glioma cells with the inhibition of Aurora A and B have revealed G2/M lagging and caspase-related cell death^42^. On the other hand, the transcription factor, FOXM1, which is known as human proto-oncogene, can maintain the activity of glioma stem cells (GSCs) and can promote the activity of β-catenin to regulate Wnt target gene expression in GSCs^43^. FOXM1 can also interact with MELK to regulate GSC mitosis^44^. FOXM1 can activate the STAT3 signaling pathway to enhance the self-renewing and tumorigenesis of GSCs^45^.

In the protein–protein interaction network, we found possible associations among SGO2, Aurora B kinase, and FOXM1, but the exact roles of these proteins in the functioning of GSCs remain unknown. To know their relationship will be helpful to understand the mechanism of GSC tumorigenicity and identifying new treatment targets in patients with glioblastomas. In budding yeast *Saccharomyces pombe*, SGO2 has been found to be localized at subtelomeres to form a chromatin domain during the G2 phase. Furthermore, SGO2 can regulate the expression of genes and cell replication timing localized at subtelomeres^34^. In our current study, the downregulation of Aurora B kinase and FOXM1 might be mediated through the gene regulation by SGO2. Further studies assessing whether SGO2 has another chromosomal localization during the cell cycle in glioblastoma cells and the relationship between *SGO2* and gene expression in glioblastoma are areas of future research.

This study had several limitations. First, it was difficult to collect a large sample of non-tumor brain tissue and low-grade human gliomas to validate *SGO2* expression. We performed a large-scale analysis of GEO profiles, TCGA, and CGGA data sets, to reveal that *SGO2* is a biomarker related to WHO pathological grading and survival outcome. We further confirmed the data analyzed from the three independent cohort studies though wet lab approaches, such as qRT-PCR and Western blot. Second, the true value of *SGO2* in the prediction of survival outcomes in patients with gliomas should be further investigated. Third, more research efforts should be invested in investigating detailed mechanisms to explain the influence of *SGO2* on the survival rates of patients with gliomas.

In conclusion, this is the first study investigating the relationship between *SGO2*, which is a conserved centromeric protein, and gliomas. *SGO2* expression revealed a positive correlation with WHO pathological grades of gliomas. Higher *SGO2* expression was associated with worse survival outcomes in patients with high-grade gliomas. Regulation of *SGO2* signal interfere the expression of mitosis related protein AURKB and FOXM1. Thus, we suggest that *SGO2* is not only a potential biomarker for disease prediction in patients with gliomas, but also has potential to be the new target of glioma treatment.

## Supplementary Information

Supplementary Figures.
