# Supplementary material for: Shugoshin 2 is a biomarker for pathological grading and survival prediction in patients with gliomas
Source: Sci Rep. 2021 Sep 17;11:18541. doi: 10.1038/s41598-021-97119-4 (PMC8448842; doi:10.1038/s41598-021-97119-4)
Supplement: Supplementary file 1 — Supplementary Figures. [file 41598_2021_97119_MOESM1_ESM.docx]

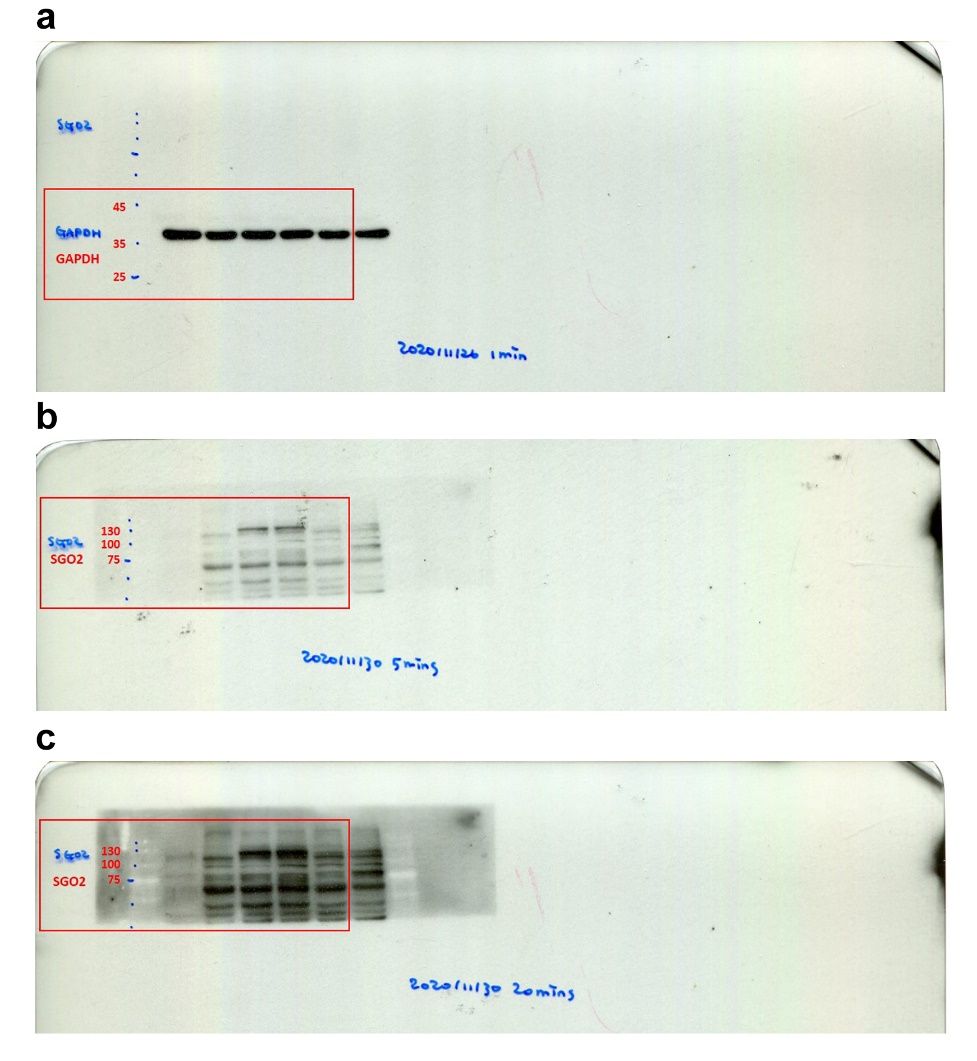


**Supplementary Fig.1 Validation of SGO2 protein levels in glioma cell lines and normal brain tissue.** Protein lysates of normal brain tissue, U87MG, LN229, GBM8401, and U118MG were applied to one SDS-PAGE. After gel electrophoresis and protein transferring, we cut the membrane as two parts according to molecular size. Anti-GADPH with 5% non-fat milk (a) and anti-SGO2 diluted at a ratio of 1:1000 with SignalBoost Immunoreaction Enhancer Kit (b and c) were incubated. GAPDH served as a loading control.


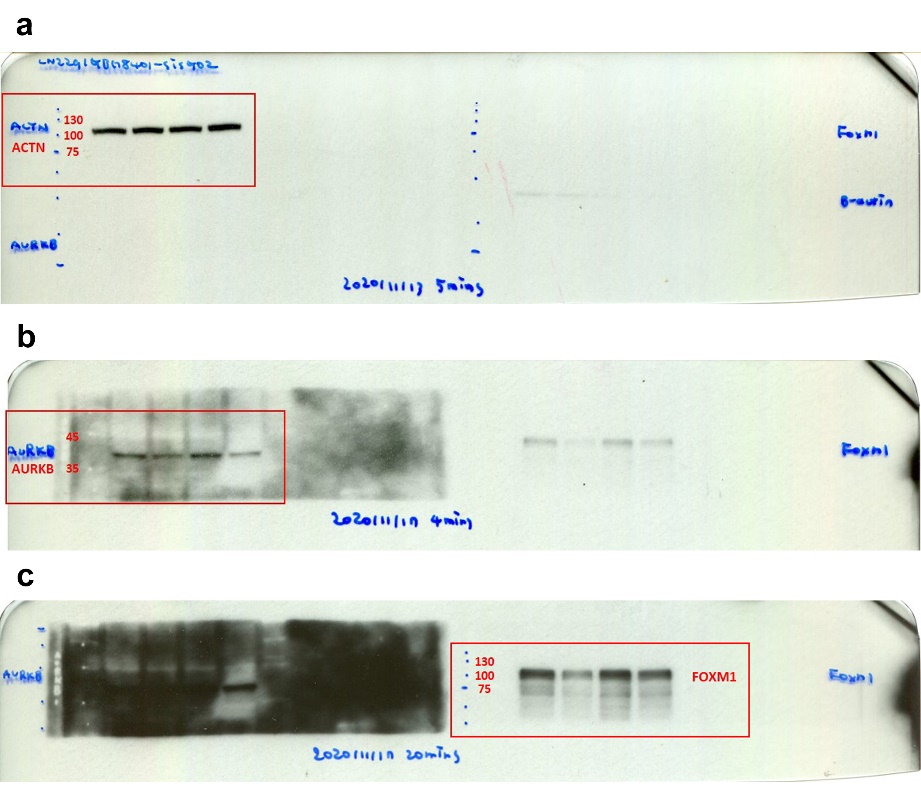


**Supplementary Fig.2 The SGO2 protein-protein interaction (PPI) network.** Protein lysates of LN229 and GBM8401 after RNA interference were applied to one SDS-PAGE. After gel electrophoresis and protein transferring, we cut the membrane as two parts according to molecular size. Anti-α-actinin with 5% non-fat milk(a) and anti-AURKB and anti-FOXM1 diluted at a ratio of 1:1000 with SignalBoost Immunoreaction Enhancer Kit (b and c) were incubated. α-actinin served as a loading control.
